# SARS-CoV-2 Infection Enhances Humoral Immune Response in Vaccinated Liver Transplant Recipients

**DOI:** 10.3390/antib13030078

**Published:** 2024-09-23

**Authors:** Jan Basri Adiprasito, Tobias Nowacki, Richard Vollenberg, Jörn Arne Meier, Florian Rennebaum, Tina Schomacher, Jonel Trebicka, Julia Fischer, Eva U. Lorentzen, Phil-Robin Tepasse

**Affiliations:** 1 Department of Medicine B for Gastroenterology, Hepatology, Endocrinology and Clinical Infectiology, University Hospital Münster, 48149 Muenster, Germany; janbasri.adiprasito@ukmuenster.de (J.B.A.); tobias.nowacki@ukm-mhs.de (T.N.); richard.vollenberg@ukmuenster.de (R.V.); joernarne.meier@ukmuenster.de (J.A.M.); florian.rennebaum@ukmuenster.de (F.R.); tina.schomacher@ukmuenster.de (T.S.); jonel.trebicka@ukmuenster.de (J.T.); julia.fischer2@ukmuenster.de (J.F.); 2 Department of Internal Medicine and Gastroenterology, Marienhospital Steinfurt, 48565 Steinfurt, Germany; 3 Institute of Virology, University Hospital Muenster, 48149 Muenster, Germany; eva.lorentzen@ukmuenster.de

**Keywords:** liver transplantation, SARS-CoV-2 infection, vaccination, humoral immunity, surrogate virus neutralization test

## Abstract

In the spring of 2020, the SARS-CoV-2 pandemic presented a formidable challenge to national and global healthcare systems. Immunocompromised individuals or those with relevant pre-existing conditions were particularly at risk of severe coronavirus disease 2019 (COVID-19). Thus, understanding the immunological processes in these patient groups is crucial for current research. This study aimed to investigate humoral immunity following vaccination and infection in liver transplant recipients. Humoral immunity analysis involved measuring IgG against the SARS-CoV-2 spike protein (anti-S IgG) and employing a surrogate virus neutralization test (sVNT) for assessing the hACE2 receptor-binding inhibitory capacity of antibodies. The study revealed that humoral immunity post-vaccination is well established, with positive results for anti-S IgG in 92.9% of the total study cohort. Vaccinated and SARS-CoV-2-infected patients exhibited significantly higher anti-S IgG levels compared to vaccinated, non-infected patients (18,590 AU/mL vs. 2320 AU/mL, *p* < 0.001). Additionally, a significantly elevated receptor-binding inhibitory capacity was observed in the *cPass^TM^TM* sVNT (96.4% vs. 91.8%, *p* = 0.004). Furthermore, a substantial enhancement of anti-S IgG levels (*p* = 0.034) and receptor-binding inhibition capacity (*p* < 0.001) was observed with an increasing interval post-transplantation (up to 30 years), calculated by generalized linear model analysis. In summary, fully vaccinated liver transplant recipients exhibit robust humoral immunity against SARS-CoV-2, which significantly intensifies following infection and with increasing time after transplantation. These findings should be considered for booster vaccination schemes for liver transplant recipients.

## 1. Introduction

The SARS-CoV-2 pandemic, which unfolded at the beginning of 2020, stands out as the most formidable medical challenge of the 21st century for both national and international healthcare systems [1]. The manifestations of this infection exhibit considerable diversity, with elderly individuals, immunocompromised patients, and those with comorbidities being particularly susceptible to SARS-CoV-2 infection and severe disease [2]. A significant proportion of liver transplant recipients who were infected developed severe COVID-19, necessitating intensive medical care [3]. Consequently, international vaccination strategies have accorded the highest priority to these and other vulnerable populations.

For the primary immunization, three antigen contacts are recommended in the current guidelines (Epid. Bull., 02/2024, STIKO, Germany). Any additional vaccination is regarded as a booster shot and is solely obligatory for particular demographic groups, including individuals aged 60 years and above, residents of care facilities, and those aged six months and above with an underlying disease that is associated with an elevated risk of a severe outcome from SARS-CoV-2 infection or a compromised immune response to the virus. Previous research indicates diminished humoral immunity after SARS-CoV-2 vaccination in liver transplant recipients compared to the general population after two vaccinations [4,5]. Factors such as age, current immunosuppressive drug regimen, leucopenia, and the time between the first vaccination and liver transplantation have been identified as crucial in this context [5,6,7]. Administering a third and fourth booster vaccination significantly enhances immunity at both the cellular and humoral levels, albeit remaining below that of healthy individuals [6]. Furthermore, it is well-established that an infection with SARS-CoV-2, following the completion of primary immunization, induces an additional boost to humoral immunity. This effect has been demonstrated in a large cohort of mostly healthy subjects, without specifically addressing individuals with underlying immunosuppressive therapy post-organ transplantation [8]. Hybrid immunity resulting from a combination of infection and vaccination has been shown to provide robust, durable protection from severe disease or hospitalization [9]. It is imperative to further elucidate the effect of immunosuppressive therapy, as it may impact the decision regarding the necessity of additional vaccinations in immunosuppressed patients.

This study aimed to investigate the humoral immune response after vaccination and SARS-CoV-2 infection in liver transplant recipients and to identify relevant influencing factors.

## 2. Materials and Methods

### 2.1. Sample Acquisition

Serum and lithium heparin samples were obtained from patients following liver transplantation between May and October 2022. The blood samples were collected after informing the patients and obtaining their written consent during their routine visits to the liver transplant outpatient clinic at Münster University Hospital. This procedure adhered to the principles of the Declaration of Helsinki and received ethical approval from the Ethics Committee of the University Hospital Münster (protocol code 2020-566-f-S).

### 2.2. Assessment of Humoral Immunity

To identify individuals with previous SARS-CoV-2 infection, the presence of IgG antibodies targeting the nucleocapsid (N) antigen (anti-N IgG) was qualitatively assessed in all serum samples. This analysis was conducted using the commercially available chemiluminescence microparticle assay (CMIA) *SARS-CoV-2 IgG* (Abbott Diagnostics, Wiesbaden, Germany). To evaluate the humoral response following SARS-CoV-2 vaccination and/or infection, IgG antibodies against the receptor-binding domain (RBD) of the spike protein subunit S1 were quantified using the SARS-CoV-2 IgG II Quant CMIA (Abbott Diagnostics). Values at or above the cutoff set at 50.0 arbitrary units (AU)/mL indicate seropositivity, with 1 AU/mL corresponding to 7.1 binding antibody units (BAU) according to the WHO. The linear range of quantification spans up to 40.000 AU/mL. All assays were executed following the manufacturer’s instructions on an Architect platform (Abbott), as detailed in previous studies [10,11,12].

For the functional characterization of serum antibodies in terms of their ability to impede the interaction between the RBD and the human host cell receptor protein angiotensin-converting enzyme 2 (hACE2), and consequently prevent cell entry and, thus, infection, the cPass^TM^ SARS-CoV-2 Neutralization Antibody Detection Kit (GenScript Biotech, Leiden, The Netherlands) was employed. In short, this assay determines hACE2-RBD binding inhibition by human sera in a blocking ELISA format [13]. The assay can be conducted rapidly in a routine diagnostic setting (biosafety level 2 conditions) and has demonstrated high reproducibility and good correlation with both conventional live virus- and pseudo-virus-based virus neutralization assays [14]. Samples, finally diluted 1:20, were tested in technical duplicates as recommended by the manufacturer. Inhibition was calculated as 1 − (OD value of the sample/OD value of the negative control) × 100%. GenScript Biotech has set the cut-off value for the CE/IVD version of the assay at 30% inhibition.

### 2.3. Statistics

Numerical variable distribution was assessed using the Shapiro–Wilk test. Depending on distribution, Student’s t-test or Mann–Whitney U test was used to evaluate differences between groups. Differences between categorical variables were assessed using the Chi Square or Fisher’s exact test, depending on sample size. General linear regression models were used to correlate variable relationships. A *p*-value of <0.05 was determined to be statistically significant. R version 4.1.2 (R Foundation for Statistical Computing, Vienna, Austria) was used for statistical analysis.

## 3. Results

### 3.1. Patient Characteristics

In this study, a total of 98 participants were recruited, with 55 males (56%) and a median age of 60.5 years. A total of 36 patients had a history of SARS-CoV-2 infection, while 62 patients had no documented history of infection. Regarding comorbidities, 34 patients (34.7%) presented with chronic renal insufficiency, 22 (22.4%) had diabetes mellitus, and 17 (17.3%) had malignancy upon study enrollment. Thirteen individuals underwent a secondary liver transplantation. Immunosuppression strategies were diverse, with 17 patients on monotherapy, 58 on dual therapy, and 23 on triple therapy. Tacrolimus (81.6%), mycophenolate mofetil (55.1%), everolimus (31.6%), and cortisone (26.5%) were the most frequently prescribed medications. The indications for liver transplantation and further details can be found in Table 1.

Vaccination distribution per patient is as follows: one vaccination for 1 patient, two vaccinations for 9 patients, three vaccinations for 42 patients, and four vaccinations for 43 patients. Three patients received a total of five vaccinations each (Table 2). The mRNA BNT162b2 vaccine was the most frequently administered. There was no significant difference in the overall vaccination history between patients with history of SARS-CoV-2 infection and those who have not experienced an infection. The details are displayed in Table 2.

### 3.2. SARS-CoV-2 Infection Augments the Humoral Immune Response in Vaccinated Liver Transplant Recipients

**Figure 1:**
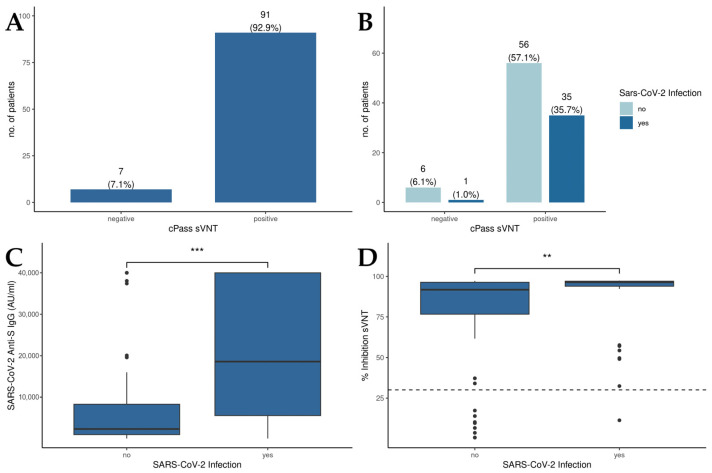
Humoral immunity in patients after liver transplantation. (**A**) Qualitative results of the *cPass^TM^* sVNT; cutoff: 30% inhibition. (**B**) Comparison of the *cPass^TM^* sVNT results between patients with or without a history of SARS-CoV-2 infection. (**C**) Detection of anti-S IgG levels in patients in with and without a history of SARS-CoV-2 infection by the Abbott CMIA. (**D**) Inhibition of RBD-hACE2 binding by sera of patients with and without a history of SARS-CoV-2 infection as measured by *cPass^TM^* sVNT; cutoff: 30% inhibition. Mann–Whitney U test was used to evaluate differences between groups. ** *p* < 0.01; *** *p* < 0.001.

The results of the *cPass*^TM^ sVNT revealed that 92.9% of all patients displayed RBD-hACE2 binding inhibitory antibodies above the cutoff value, suggesting an effective induction of neutralizing antibodies against SARS-CoV-2 through vaccination (Figure 1A). Moreover, no significant differences in *cPass^TM^* sVNT inhibition levels between patients with a history of SARS-CoV-2 infection and the overall patient population could be detected (Figure 1B). Concerning anti-S IgG levels (Figure 1C), those patients who experienced a SARS-CoV-2 infection displayed significantly higher anti-S IgG levels compared to uninfected individuals (18,590 AU/mL vs. 2320 AU/mL, *p* < 0.001). Examining the percentage of inhibition derived from the *cPass^TM^* sVNT (Figure 1D), liver transplant recipients with a history of SARS-CoV-2 infection demonstrated a notably higher inhibition capacity than those without prior infection (96.4% vs. 91.8%, *p* = 0.004).

### 3.3. Time-Dependent Amplification of Humoral Immune Response in Liver Transplant Recipients: A Linear Model Analysis

**Figure 2:**
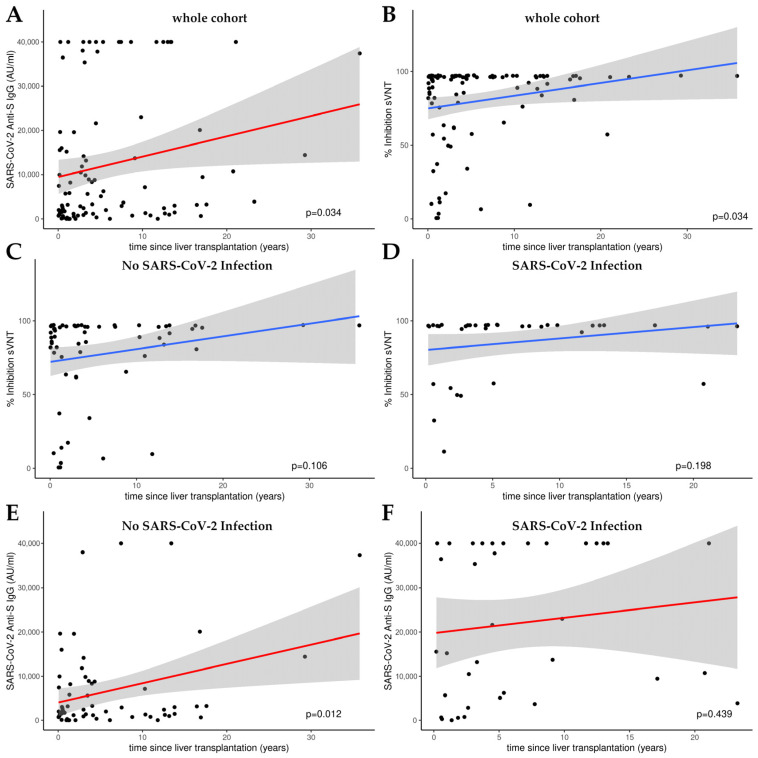
Linear model (LM) plots illustrating the humoral immune response over time, since liver transplantation. (**A**) Increase in anti-S IgG levels (AU/mL) with increasing time post-transplantation. (**B**) Increase in relative hACE2 binding inhibition percentage, indicative of enhanced viral neutralization capacity over time following transplantation. Inhibition over time in patients without (**C**) and with (**D**) a history of SARS-CoV-2 infection as determined by *cPass^TM^* sVNT. Anti-S IgG levels over time in patients without (**E**) and with (**F**) a history of SARS-CoV-2 infection as measured by CMIA. Cutoff value *cPass^TM^* sVNT: 30% inhibition; the linear range of the anti-S CMIA ends at 40,000 AU/mL.

Figure 2 presents the outcomes of linear model (LM) plots illustrating the temporal dynamics of the humoral immune response following liver transplantation. A significant rise in anti-S IgG levels and the percentage of hACE2 binding inhibition were evident with the progression of time post-transplantation, signifying a time-dependent amplification of humoral immunity (Figure 2A,B).

When analyzing the sub-cohorts with or without SARS-CoV-2 infection, a significant increase in humoral immunity was only observed for anti-S IgG levels in the group of patients who had not been infected (*p* = 0.012; see Figure 2E). There was no significant increase in the percentage of hACE2 binding inhibition in either subgroup (see Figure 2C,D,F).

## 4. Discussion

An infection with SARS-CoV-2 can lead to an individually varying disease course, ranging from mild to severe outcomes, including fatalities. Vaccination against SARS-CoV-2 has led to a significant reduction in severe disease progression. Patients with underlying conditions such as diabetes, hypertension, heart disease, and tumor-related illnesses, especially, have benefited from vaccination. Another particularly vulnerable patient group includes organ transplant recipients, where a substantial risk reduction by vaccination has also been demonstrated [15,16]. However, throughout the vaccination campaigns, it has become evident that for sustained and adequate humoral immunity, as well as protection against severe disease outcomes, a primary immunization with two doses is insufficient, because, over time, antibody levels in the blood decrease [8]. Consequently, booster vaccinations are recommended [15,17,18]). Furthermore, as described before, it is well-established that an infection with SARS-CoV-2, following the completion of primary immunization, induces an additional boost to humoral immunity [9]. This effect has been demonstrated in a large cohort, primarily comprising healthy subjects, without specifically addressing individuals with underlying immunosuppressive therapy after organ transplantation [8]. Regarding liver transplant patients, the current state of research is inconclusive. Individual studies suggest a booster effect on humoral and cellular immunity of a SARS-CoV-2 infection as compared to vaccination alone in solid organ transplant recipients [19]. Humoral immunity was determined in this study by determining SARS-CoV-2-specific IgG and IgM without respect to the neutralization capacity of the antibodies. Interestingly, humoral immunity against SARS-CoV-2 was shown to wane more within the first year after infection in comparison to individuals without immunosuppression [20]. Additionally, infection with SARS-CoV-2 leads to lower levels of anti-nucleocapsid IgG in liver transplant patients at 3 and 6 months and to greater declines in humoral immunity over time compared to healthy controls [21]. Clarifying this in more detail is clinically highly relevant, because the increasing diversity in patient cohorts as to the number of booster vaccinations and past infections, along with the individually varying timing of infections and vaccinations, complicates the decision-making for additional vaccinations.

In this study, we demonstrate that the effect of hybrid immunity on humoral immunity observed in healthy subjects also extends to patients undergoing immunosuppressive medication after liver transplantation. Our study utilized SARS-CoV-2 anti-S IgG levels and the neutralizing capacities of antibodies as measured by *cPass^TM^* sVNT as parameters for assessing humoral immunity in liver transplant recipients after vaccination and SARS-CoV-2 infection. These parameters were significantly higher after both vaccination and infection compared to vaccination alone. Notably, this effect persisted even though most participants had already received two vaccine doses and at least one booster (third vaccination) at the time of blood collection. In conclusion, an infection in liver transplant recipients between two booster shots should be taken into consideration when planning the next booster vaccination. Current guidelines recommend annual booster vaccination for individuals aged 60 years and above, residents of care facilities, and those aged six months and above with an underlying disease that is associated with an elevated risk of a severe outcome from SARS-CoV-2 infection or a compromised immune response to the virus (Epid. Bull., 02/2024, STIKO, Germany). Whether the one-year interval is sufficient for liver transplant recipients after infection needs to be addressed in further studies.

Another crucial aspect highlighted by our study is the time-dependent increase in humoral immunity with an increasing interval from transplantation, as measured by the percentage of inhibition in the *cPass^TM^* sVNT and of SARS-CoV-2 anti-S IgG levels. The diminished response to vaccinations post-transplantation is also documented for other pathogens such as hepatitis B [22,23]. We were not able to show this relationship between time after liver transplantation and increasing humoral immunity for the subgroups (SARS-CoV-2 infection vs. no SARS-CoV-2 infection), possibly due to the limited number of data points in the subgroups. Further studies are necessary for better representation and comparison of these subgroups in this regard. A contributing factor to the reduced immune response following vaccinations within the first year post-transplantation can be attributed to the administration of more potent immunosuppressive drug regimens. Current guidelines recommend a dual immunosuppressive therapy, supplemented with prednisolone as a third immunosuppressive medication during the initial months post-transplantation to prevent rejection reactions.

In addition to the small number of patients already mentioned, the limitations of this study lie in its focus on humoral immunity. A supplementary presentation of cellular immunity, in particular with regard to the infection status and the vaccination status of the patients, could provide additional insights into the optimal vaccination regimen for immunocompromised patients. Moreover, linear modeling is of limited applicability due to the 40,000 AU/mL cutoff for anti-S CMIA, as values above this threshold may be underestimated. Furthermore, the absence of data concerning the progression and severity of the disease, as well as the specific SARS-CoV-2 variants, precludes the establishment of a correlation between the aforementioned points of humoral immunity in liver transplant recipients. Future studies should prioritize this area of investigation.

## 5. Conclusions

Our study demonstrates that SARS-CoV-2 infection after vaccination results in an increase in SARS-CoV-2 anti-S IgG levels and the percentage of hACE2 binding inhibitory antibodies in liver transplant recipients. The extent of both humoral immunity parameters is time-dependent and intensifies with increasing time after liver transplantation. The data presented emphasizes the importance of monitoring humoral immune responses in vaccinated liver transplant recipients, particularly in the context of SARS-CoV-2 infection and post-transplantation duration. By understanding the dynamics of immune enhancement following infection and over time, healthcare providers can customize vaccination strategies to optimize immune protection and clinical outcomes in this unique patient population. Studying the long-term immune responses, durability of protection, and optimal vaccination intervals in liver transplant recipients following SARS-CoV-2 infection can provide valuable insights for evidence-based recommendations on personalized vaccination strategies and clinical management.

## Figures and Tables

**Table 1:** Cohort characteristics of liver transplant recipients.

		No SARS-CoV-2 Infection (*n* = 62)	SARS-CoV-2 Infection (*n* = 36)	*p*-Value
Patient characteristics	Age, years median (IQR)	62.5 (17.8)	60.5 (15.5)	0.563
	Sex, male, total	35 (35.7%)	20 (20.4%)	0.931
	Months after transplantation, median (IQR)	36.7 (111)	54.6 (92)	0.218
	Retransplantation	7 (7.1%)	6 (6.1%)	0.449
Pre-existing conditions	Diabetes mellitus	14 (14.2%)	8 (8.2%)	0.140
	Kidney insufficiency	24 (24.5%)	10 (10.2%)	0.273
	Inflammatory disease	16 (16.3%)	12 (12.2%)	0.427
Medication	Tacrolimus	50 (51%)	30 (30.6%)	0.740
	Everolimus	19 (19.4%)	12 (12.2%)	0.783
	Mycophenolatmofetil	36 (36.7%)	18 (18.4%)	0.439
	Ciclosporin	3 (3.1%)	2 (2%)	0.876
	Prednisolon	17 (17.3%)	9 (9.2%)	0.794
	Sirolimus	2 (2%)	2 (2%)	0.574
	Immunosuppressive therapy			0.957 ^1^
	Immunosuppressive monotherapy	11 (11.2%)	6 (6.1%)	0.892
	Immunosuppressive dual therapy	36 (36.7%)	22 (22.4%)	0.767
	Immunosuppressive triple therapy	15 (15.3%)	8 (8.2%)	0.824
Indication for liver transplantation				0.993 ^1^
	Hepatocellular carcinoma	7 (7.1%)	10 (10.2%)	0.676
	Primary sclerosing cholangitis	6 (6.1%)	6 (6.1%)	1
	Secondary sclerosing cholangitis	1 (1%)	2 (2%)	0.901
	Autoimmune hepatitis	2 (2%)	4 (4.1%)	0.858
	Ethanol related	4 (4.1%)	9 (9.2%)	0.632
	Drug induced liver injury	2 (2%)	3 (3.1%)	0.876
	Viral hepatitis	3 (3.1%)	4 (4.1%)	0.727
	Budd Chiari	1 (1%)	1 (1%)	1
	Non-alcoholic steatohepatitis	1 (1%)	4 (4.1%)	0.426
	Cryptogene	6 (6.1%)	10 (10.2%)	0.945
	Other	3 (3.1%)	9 (9.2%)	0.544

^1^ Fisher’s exact test results for comparison of the whole cohort.

**Table 2:** Vaccination data of liver transplant patients.

		No SARS-CoV-2 Infection (*n* = 62)	SARS-CoV-2 Infection (*n* = 36)	*p*-Value
Manufacturer of first vaccine				0.727
mRNA (BNT162b2 and mRNA-1273)		58 (93.5%)	33 (91.7%)	n.d.
vector-based (ChAdOx1-S and Ad26.COV2.S)		4 (6.5%)	3 (8.3%)	n.d.
no vaccination		0	0	n.d.
Manufacturer of second vaccine				0.070
mRNA (BNT162b2 and mRNA-1273)		62 (100%)	33 (91.7%)	0.021
vector-based (ChAdOx1-S and Ad26.COV2.S)		0 (0%)	2 (5.6%)	0.061
no vaccination		0 (0%)	1 (2.8%)	0.187
Manufacturer of third vaccine				0.049
mRNA (BNT162b2 and mRNA-1273)		58 (93.5%)	29 (80.6%)	n.d.
vector-based (ChAdOx1-S and Ad26.COV2.S)		0	0	n.d.
no vaccination		4 (6.5%)	7 (19.4%)	n.d.
Manufacturer of fourth vaccine				0.040
mRNA (BNT162b2 and mRNA-1273)		34 (54.8%)	12 (33.3%)	n.d.
vector-based (ChAdOx1-S and Ad26.COV2.S)		0	0	n.d.
no vaccination		28 (45.2%)	24 (66.7%)	n.d.
Manufacturer of fifth vaccine				0.901
mRNA (BNT162b2 and mRNA-1273)		2 (3.2%)	1 (2.8%)	n.d.
vector-based (ChAdOx1-S and Ad26.COV2.S)		0	0	n.d.
no vaccination		60 (96.8%)	35 (97.2%)	n.d.
Vaccination History	vaccinations			0.101
	1 vaccination	0 (0%)	1 (2.8%)	0.187
	2 vaccinations	3 (4.8%)	6 (16.7%)	0.051
	3 vaccinations	25 (40.3%)	17 (47.2%)	0.506
	4 vaccinations	32 (51.6%)	11 (30.6%)	0.043
	5 vaccinations	2 (3.2%)	1 (2.8%)	0.901

## Data Availability

Original data are unavailable due to patient’s privacy.

## List of Abbreviations

**Abbreviation**	**Definition**
AU	arbitrary units
BAU	binding antibody units
CMIA	chemiluminescence microparticle assay
ELISA	enzyme-linked immunosorbent assay
hACE2	human angiotensin-converting enzyme 2
RBD	receptor-binding domain
sVNT	surrogate virus neutralization test

## References

[1] Wu J.T., Leung K., Leung G.M. (2020). Nowcasting and forecasting the potential domestic and international spread of the 2019-nCoV outbreak originating in Wuhan, China: A modelling study. Lancet.

[2] Wu Z., McGoogan J.M. (2020). Characteristics of and Important Lessons from the Coronavirus Disease 2019 (COVID-19) Outbreak in China: Summary of a Report of 72 314 Cases from the Chinese Center for Disease Control and Prevention. JAMA.

[3] Imam A., Abukhalaf S.A., Merhav H., Abu-Gazala S., Cohen-Arazi O., Pikarsky A.J., Safadi R., Khalaileh A. (2020). Prognosis and Treatment of Liver Transplant Recipients in the COVID-19 Era: A Literature Review. Ann. Transplant..

[4] Thuluvath P.J., Robarts P., Chauhan M. (2021). Analysis of antibody responses after COVID-19 vaccination in liver transplant recipients and those with chronic liver diseases. J. Hepatol..

[5] Ruether D.F., Schaub G.M., Duengelhoef P.M., Haag F., Brehm T.T., Fathi A., Wehmeyer M., Jahnke-Triankowski J., Mayer L., Hoffmann A. (2022). SARS-CoV2-specific Humoral and T-cell Immune Response After Second Vaccination in Liver Cirrhosis and Transplant Patients. Clin. Gastroenterol. Hepatol..

[6] Harberts A., Schaub G.M., Ruether D.F., Duengelhoef P.M., Brehm T.T., Karsten H., Fathi A., Jahnke-Triankowski J., Fischer L., Addo M.M. (2022). Humoral and Cellular Immune Response After Third and Fourth SARS-CoV-2 mRNA Vaccination in Liver Transplant Recipients. Clin. Gastroenterol. Hepatol..

[7] Herrera S., Colmenero J., Pascal M., Escobedo M., Castel M.A., Sole-González E., Palou E., Egri N., Ruiz P., Mosquera M. (2021). Cellular and humoral immune response after mRNA-1273 SARS-CoV-2 vaccine in liver and heart transplant recipients. Am. J. Transplant..

[8] Hall V., Foulkes S., Insalata F., Kirwan P., Saei A., Atti A., Wellington E., Khawam J., Munro K., Cole M. (2022). Protection against SARS-CoV-2 after COVID-19 Vaccination and Previous Infection. N. Engl. J. Med..

[9] Bobrovitz N., Ware H., Ma X., Li Z., Hosseini R., Cao C., Selemon A., Whelan M., Premji Z., Issa H. (2023). Protective effectiveness of previous SARS-CoV-2 infection and hybrid immunity against the omicron variant and severe disease: A systematic review and meta-regression. Lancet Infect. Dis..

[10] Vollenberg R., Tepasse P.-R., Kühn J.E., Hennies M., Strauss M., Rennebaum F., Schomacher T., Boeckel G., Lorentzen E., Bokemeyer A. (2022). Humoral Immune Response in IBD Patients Three and Six Months after Vaccination with the SARS-CoV-2 mRNA Vaccines mRNA-1273 and BNT162b2. Biomedicines.

[11] Vollenberg R., Tepasse P.-R., Lorentzen E., Nowacki T.M. (2022). Impaired Humoral Immunity with Concomitant Preserved T Cell Reactivity in IBD Patients on Treatment with Infliximab 6 Month after Vaccination with the SARS-CoV-2 mRNA Vaccine BNT162b2: A Pilot Study. J. Pers. Med..

[12] Schoefbaenker M., Neddermeyer R., Guenther T., Mueller M.M., Romberg M.-L., Classen N., Hennies M.T., Hrincius E.R., Ludwig S., Kuehn J.E. (2023). Surrogate Virus Neutralisation Test Based on Nanoluciferase-Tagged Antigens to Quantify Inhibitory Antibodies against SARS-CoV-2 and Characterise Omicron-Specific Reactivity in a Vaccination Cohort. Vaccines.

[13] Tan C.W., Chia W.N., Qin X., Liu P., Chen M.I.-C., Tiu C., Hu Z., Chen V.C.-W., Young B.E., Sia W.R. (2020). A SARS-CoV-2 surrogate virus neutralization test based on antibody-mediated blockage of ACE2-spike protein-protein interaction. Nat. Biotechnol..

[14] Taylor S.C., Hurst B., Charlton C.L., Bailey A., Kanji J.N., McCarthy M.K., Morrison T.E., Huey L., Annen K., DomBourian M.G. (2021). A New SARS-CoV-2 Dual-Purpose Serology Test: Highly Accurate Infection Tracing and Neutralizing Antibody Response Detection. J. Clin. Microbiol..

[15] Fiolet T., Kherabi Y., MacDonald C.-J., Ghosn J., Peiffer-Smadja N. (2022). Comparing COVID-19 vaccines for their characteristics, efficacy and effectiveness against SARS-CoV-2 and variants of concern: A narrative review. Clin. Microbiol. Infect..

[16] Haas E.J., Angulo F.J., McLaughlin J.M., Anis E., Singer S.R., Khan F., Brooks N., Smaja M., Mircus G., Pan K. (2021). Impact and effectiveness of mRNA BNT162b2 vaccine against SARS-CoV-2 infections and COVID-19 cases, hospitalisations, and deaths following a nationwide vaccination campaign in Israel: An observational study using national surveillance data. Lancet.

[17] Naaber P., Tserel L., Kangro K., Sepp E., Jürjenson V., Adamson A., Haljasmägi L., Rumm A.P., Maruste R., Kärner J. (2021). Dynamics of antibody response to BNT162b2 vaccine after six months: A longitudinal prospective study. Lancet Reg. Health Eur..

[18] Andrews N., Stowe J., Kirsebom F., Toffa S., Sachdeva R., Gower C., Ramsay M., Lopez Bernal J. (2022). Effectiveness of COVID-19 booster vaccines against COVID-19-related symptoms, hospitalization and death in England. Nat. Med..

[19] Ayala-Borges B., Escobedo M., Egri N., Herrera S., Crespo M., Mirabet S., Arias-Cabrales C., Vilella A., Palou E., Mosquera M.M. (2023). Impact of SARS-CoV-2 Infection on Humoral and Cellular Immunity in a Cohort of Vaccinated Solid Organ Transplant Recipients. Vaccines.

[20] Kirchner T., Heinrich S., Bonifacius A., Engel B., Ruhl L., Pink I., Thomas N., Martens J., Hoeper M.M., Blasczyk R. (2022). Reduced humoral but stable cellular SARS-CoV-2-specific immunity in liver transplant recipients in the first year after COVID-19. PLoS ONE.

[21] Caballero-Marcos A., Salcedo M., Alonso-Fernández R., Rodríguez-Perálvarez M., Olmedo M., Graus Morales J., Cuervas-Mons V., Cachero A., Loinaz-Segurola C., Iñarrairaegui M. (2021). Changes in humoral immune response after SARS-CoV-2 infection in liver transplant recipients compared to immunocompetent patients. Am. J. Transplant..

[22] van Thiel D.H., el-Ashmawy L., Love K., Gavaler J.S., Starzl T.E. (1992). Response to hepatitis B vaccination by liver transplant candidates. Dig. Dis. Sci..

[23] Loinaz C., de Juanes J.R., Gonzalez E.M., López A., Lumbreras C., Gómez R., Gonzalez-Pinto I., Jiménez C., Garcia I., Fuertes A. (1997). Hepatitis B vaccination results in 140 liver transplant recipients. Hepatogastroenterology.

